# Does an educational website improve psychological outcomes and satisfaction among family members of intensive care unit patients?

**DOI:** 10.5935/2965-2774.20230113-en

**Published:** 2023

**Authors:** Tarissa da Silva Ribeiro Haack, Regis Goulart Rosa, Cassiano Teixeira, Daniel Sganzerla, Caroline Cabral Robinson, Cláudia Severgnini Eugênio, Cleidilene Ramos Magalhães

**Affiliations:** 1 Postgraduate Program in Health Education, Universidade Federal de Ciências da Saúde de Porto Alegre - Porto Alegre (RS), Brazil; 2 Research Projects Office, Hospital Moinhos de Vento - Porto Alegre (RS), Brazil; 3 Postgraduate Program in Rehabilitation Sciences, Universidade Federal de Ciências da Saúde de Porto Alegre - Porto Alegre (RS), Brazil; 4 Universidade Federal de Ciências da Saúde de Porto Alegre - Porto Alegre (RS), Brazil

**Keywords:** Health information systems, Internet, Anxiety, Depression, Family, Personal satisfaction, Outcome assessment, health care, Intensive care units

## Abstract

**Objective:**

To evaluate the impact of an educational website on satisfaction and symptoms of anxiety and depression among family members of critically ill adult patients.

**Methods:**

We embedded an analysis of website access in a cohort study conducted in intensive care units with flexible visiting hours in Brazil. Family members were guided to access an educational website designed to help them understand the processes and emotions associated with an intensive care unit stay. Subjects were evaluated for baseline data within the first 48 hours following enrollment and outcome assessment at up to 7 days after patient discharge from the intensive care unit, death, or until the 30^th^ day of the study. The main outcomes were satisfaction using the Critical Care Family Needs Inventory and the presence of anxiety and depression symptoms using the Hospital Anxiety and Depression Scale.

**Results:**

A total of 532 family members were evaluated during the study period. Of these, 61 (11.5%) accessed the website. After adjustments, family members who accessed the website had significantly better mean Critical Care Family Needs Inventory scores (152.8 *versus* 145.2, p = 0.01) and a lower prevalence of probable clinical anxiety (prevalence ratio 0.35; 95%CI 0.14 - 0.89) than family members who did not access the website. There were no differences regarding symptoms of depression.

**Conclusion:**

Access to an educational website was associated with higher family satisfaction with care and a lower prevalence of clinical anxiety.

## INTRODUCTION

Critical illness of a close relative is often a traumatic moment in life and can cause great distress.^(1,2,3,4,5,6)^ In addition, the ambience of an intensive care unit (ICU) is often perceived as unwelcoming.^(7,8,9,10)^ The cold behavior of ICU staff^(11)^ and the medical jargon frequently used to explain complex diseases^(1)^ can worsen the daily routine interaction between ICU staff and patients’ relatives.^(12,13,14)^ These breakdowns in communication may contribute to long-term symptoms of psychological distress among relatives.^(5,15,16,17)^ These relatives may also witness invasive and unfamiliar medical procedures and devices.^(5,18)^ In addition, family members are often asked to act as surrogate decision-makers when subjects are temporarily or permanently incapacitated.^(12)^

Aiming to resolve this dilemma and their doubts, relatives often turn to inadequate channels (e.g., word of mouth, television/cinema, and internet) that produce unreliable or useless information about the subject’s situation, creating improbable expectations.^(19,20)^ Therefore, the search for health information is one of the most common reasons that drives people to use the internet because they feel poorly informed.^(21,22)^

The internet can be used for the recovery of critical care survivors through web-based intensive care recovery programs emphasizing mental health improvement of the patients.^(23)^ Previously, a website and an information brochure designed to meet relatives’ needs improved family members’ comprehension about ICU patient aspects and recovery and reduced their prevalence of stress symptoms.^(18)^

Therefore, the aim of the present study was to investigate the effects of an educational website on satisfaction and symptoms of depression and anxiety among family members of critically ill patients in the context of flexible ICU visiting hours.

## METHODS

**Figure 1:**
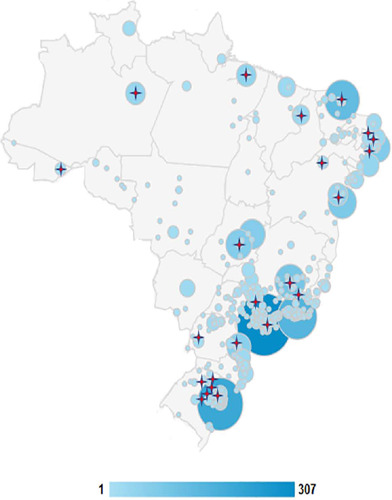
Website access map

We embedded an analysis about website access in a multicenter longitudinal cohort study nested in a clusterrandomized crossover trial (ICU visits study) conducted from April 2017 to June 2018 in 35 ICUs with flexible visiting hours in Brazil^(24)^ (Figure 1). Inclusion criteria were the closest relatives of critically ill patients who were cluster randomized to a flexible family visitation model (up to 12 hours/day) during the study. The exclusion criterion was communication difficulty (did not speak Portuguese, limitations to answering the self-administered questionnaire such as illiteracy, uncorrected visual and/or hearing impairment). The institutional review boards of all participating centers approved the study, and written consent was obtained from all participant family members. The study follows the STROBE statement (Supplementary Material).

Stars represent study centers. Of the 35 intensive care units allocated in the study, 12 had no access to the website by family members. The legend represents the intensive care units invited to participate in the initial study.^(24)^

### Interventions

The flexible visitation model was composed of two parts: (a) the flexibilization of ICU visiting hours, in which one or two close family members were allowed to visit the subject for up to 12 hours/day in addition to meeting the eligibility criteria for the study, and (b) family education, in which these family members had to attend at least one structured meeting where they received education about the ICU environment, common procedures, multidisciplinary work, infection control, palliative care, and *delirium*.^(24)^

Additionally, family members had access to an information brochure and website designed to help them understand the various processes and emotions associated with an ICU stay and improve cooperation without increasing ICU staff workload. The content of the tool was discussed at multidisciplinary meetings among physicians, nurses, respiratory therapists, and psychologists from the principal investigator center.

The website (www.utivisitas.com.br) was developed to meet relatives’ cognitive and emotional needs and included four domains: (a) About us, to clearly state who guarantees the website’s scientific content; (b) ICU knowledge, to describe the ICU peculiarities (staff, multidisciplinary rounds, patient care, supportive technology, and subject security); (c) Subject knowledge, to describe the critical disease (organ dysfunction, prognosis, possibility of complications during ICU stay, and rehabilitation); and (d) Visit knowledge, to describe the objectives of the flexibilization of visitation (role of social visit, security of visit, and familiar engagement).

### Outcomes and follow-up

The main study outcomes were satisfaction with care, assessed using the Critical Care Family Needs Inventory (CCFNI),^(25)^ which addresses satisfaction in 5 domains (proximity, information, reassurance, comfort, and support) with total scores ranging from 43 (worst) to 172 (best), and symptoms of anxiety and depression, assessed using the Hospital Anxiety and Depression Scale (HADS),^(26)^ with scores ranging from 0 - 21 (> 7 points indicating moderate anxiety or depression).

Data from family members who accessed the website were evaluated by researchers and compared with those of family members who did not access it. Family members were evaluated within the first 48 hours following patient enrollment for baseline data and up to 7 days after patient discharge from the ICU, death, or until the 30^th^ day of the study for outcome assessment using self-administered questionnaires.

### Sample size

The sample size, as well as the theoretical rationale, design and eligibility criteria of the study in which this cohort is nested, was previously published.^(24,27)^ In the current study, a sample of > 500 family members was evaluated, which refers to a consecutive sample of family members who participated in the original study, with no formal sample size calculation for this secondary analysis.

### Statistical analysis

Qualitative variables were described using absolute and relative frequencies, while quantitative variables were described as the mean (and standard deviation) or median (and interquartile range). The factors associated with access to the website were verified using the generalized estimating equation (GEE), with adjustment for the hospital (cluster) of origin, gender, age, years of study, initial HADS score, and vital status of the patient, using a Poisson distribution with robust estimation for variance. The prevalence ratio or mean differences were used according to the evaluated data. The evaluation of outcomes (CCFNI, anxiety-HADS, and depression-HADS scores) was adjusted for age, education, and HADS scores at baseline and patient survival status at the end of follow-up. The level of significance adopted was 5%, and the software used in the analysis was R version 3.5.1.

## RESULTS

**Figure 2:**
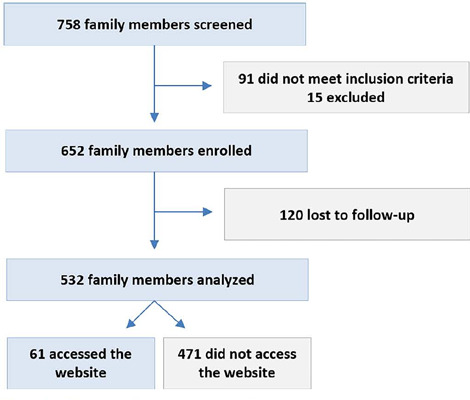
Flowchart of subjects excluded from the study

A total of 532 family members were evaluated during the study period (Figure 2). Of these, 61 (11.4%) accessed the website. The median age was 45.7 (13.5) years, 71.4% were female, and the median educational attainment was 11.4 years. Prior to their relative’s ICU admission, 14.5% and 14.4% had anxious and depressive diagnoses, respectively (Table 1).

**Table 1:** Baseline characteristics

	Total (n = 532)	Accessed the website (n = 61)	Did not access the website (n = 471)
Age (years)	45.7 ± 13.5	44.6 ± 11.4	45.9 ± 13.8
Years of education	11.6 ± 5.0	12.8 ± 5.5	11.4 ± 4.9
Household income (United States dollars)	1,235.2 (691.7 - 1,976.3)	1,334.0 (716.4 - 2,346.8)	1,235.2 (667.0 - 1,976.3)
Female gender	380/532 (71.4)	45/61 (73.8)	335/471 (71.1)
Higher education	224/466 (48.1)	28/53 (52.8)	196/413 (47.5)
Employed	275/523 (52.6)	31/60 (51.7)	244/463 (52.7)
Living with care recipient	291/523 (55.6)	37/60 (61.7)	254/463 (54.9)
History of anxiety	76/523 (14.5)	11/60 (18.3)	65/463 (14.0)
HADS anxiety subscale score > 7	204/521 (39.2)	24/60 (40.0)	180/461 (39.0)
History of depression	74/521 (14.2)	10/60 (16.7)	64/461 (13.9)
HADS depression subscale score > 7	127/520 (24.4)	19/60 (31.7)	108/460 (23.5)
Surrogate decision-makers	481/516 (93.2)	54/58 (93.1)	427/458 (93.2)
Patient death	23/532 (4.3)	1/61 (1.6)	22/471 (4.7)

HADS - Hospital Anxiety and Depression Scale. Results expressed as mean ± SD, median (interquartile range) or n/total (%).

A multivariable analysis showed that years of education (risk ratio - RR 1.06; 95% confidence interval - 95%CI 1.01 - 1.11) and HADS depression scores >7 (RR 1.74; 95%CI 1.05 - 2.90) at baseline were independently associated with website access (Table 2).

**Table 2:** Factors associated with accessing the website

Associated factor	Accessed the website	Did not access the website	Effect estimate	p value
Age (years)	44.6 ± 11.4	45.9 ± 13.8	1.00 (0.99 - 1.02)	0.92
Years of education	12.8 ± 5.5	11.4 ± 4.9	1.06 (1.01 - 1.11)	0.01
Household income (US$)	1,334.0 (716.4 - 2,346.8)	1,235.2 (667.0 - 1,976.3)	1.00 (0.99 - 1.01)	0.69
Female gender	45/380 (11.8)	16/152 (10.5)	1.38 (0.87 - 2.18)	0.17
Higher education	28/224 (12.5)	25/242 (10.3)	1.24 (0.81 - 1.91)	0.31
Employed	31/275 (11.3)	29/248 (11.7)	0.92 (0.60 - 1.42)	0.70
Living with care recipient	37/291 (12.7)	23/232 (9.9)	1.15 (0.64 - 2.07)	0.63
History of anxiety	11/76 (14.5)	49/447 (11.0)	1.38 (0.70 - 2.71)	0.35
HADS anxiety subscale score > 7	24/204 (11.8)	36/317 (11.4)	1.23 (0.75 - 2.02)	0.41
History of depression	10/74 (13.5)	50/447 (11.2)	1.31 (0.67 - 2.57)	0.43
HADS depression subscale score > 7	19/127 (15.0)	41/393 (10.4)	1.77 (1.07 - 2.91)	0.02
Surrogate decision-makers	54/481 (11.2)	4/35 (11.4)	0.91 (0.34 - 2.46)	0.85
Patient death	1/23 (4.3)	60/509 (11.8)	0.36 (0.05 - 2.62)	0.31

HADS - Hospital Anxiety and Depression Scale; United States dollar. Results expressed as mean ± mean ± SD, median (interquartile range) or n/total (%).

After adjusting for age, education, and HADS scores at baseline and patient survival status at the end of follow-up, family members who accessed the website had significantly better mean CCFNI scores (effect estimate, 6.33 [95%CI 1.44 - 11.21], p = 0.01) and lower prevalence of probable clinical anxiety (prevalence ratio, 0.35 [95%CI, 0.14 - 0.89], p = 0.003) than family members who did not access the website (Table 3). There were no significant differences between the two study groups regarding depression symptoms.

**Table 3:** Association of accessing the website with depression, anxiety, and critical care family needs

Outcomes	Accessed the website	Did not access the website	Effect estimate	p value*
**HADS**				
HADS anxiety	5.1 ± 3.6	6.2 ± 4.0	-1.27 (-2.12 - -0.43)	0.003
HADS depression	4.7 ± 3.9	4.8 ± 3.8	0.13 (-0.50 - 0.77)	0.68
Total HADS	9.8 ± 7.0	11.0 ± 7.2	-0.85 (-2.02 - 0.32)	0.15
HADS anxiety subscale > 7 (%)†	14/61 (23.0)	157/468 (33.5)	0.62 (0.41 - 0.96)	0.03
HADS depression subscale > 7 (%)†	16/61 (26.2)	117/468 (25.0)	0.98 (0.70 - 1.36)	0.89
HADS anxiety subscale > 10 (%)†	3/61 (4.9)	68/468 (14.5)	0.31 (0.12 - 0.78)	0.01
HADS depression subscale > 10 (%)†	4/61 (6.6)	39/468 (8.3)	0.80 (0.34 - 1.91)	0.62
**CCFNI**				
CCFNI satisfaction score	152.8 ± 16.2	145.2 ± 18.9	6.33 (1.44 - 11.21)	0.01
Safety score	26.3 ± 2.4	25.2 ± 3.1	1.03 (0.25 - 1.80)	0.01
Proximity score	32.8 ± 3.5	31.3 ± 4.1	1.15 (0.10 - 2.20)	0.03
Information score	29.0 ± 3.3	27.7 ± 4.0	1.08 (0.10 - 2.05)	0.03
Comfort score	19.7 ± 3.6	18.9 ± 3.6	0.74 (-0.26 - 1.74)	0.15
Support score	45.1 ± 5.8	42.0 ± 6.8	2.43 (0.74 - 4.12)	0.005
Self-perception of involvement in subject care score	17.0 ± 6.9	13.3 ± 7.0	3.86 (1.93 - 5.80)	<0.001

HADS - Hospital Anxiety and Depression Scale; CCFNI - Critical Care Family Needs Inventory. * Adjusted by age, education, HADS scores at baseline, and patient survival status at the end of follow-up. † Effect estimate was the mean difference, except prevalence ratio, according to the evaluated data. Results expressed as mean ± SD, median (interquartile range) or n/total (%).

## DISCUSSION

This study showed that access to an educational website was associated with less anxiety and greater satisfaction among family members of ICU patients during flexible visiting hours; however, this association may not be causal.

A pivotal study by Cameron et al.^(17)^ showed that a large percentage of caregivers (67% immediately after ICU discharge and 43% at 1 year) reported depressive symptoms. Regarding these findings, adequate communication between ICU practitioners and patients’ families appears essential to reduce these symptoms.^(2,4,11,15)^ Family members consider it a very important part of care to receive regular, clear information. However, they report difficulties in obtaining information and often find the information hard to understand. The agreement between the prognosis given by the physician and what the relative had understood indicated that comprehension is, in fact, an issue. In this context, alternative information skills (e.g., brochures or websites) could be associated with improvement and particularly seem to help relatives better understand medical decisions and treatment.

The stress and anxiety induced by unplanned ICU admission and the hostility of this environment may lead proxies to search for health and disease information. Therefore, the internet has become a major source of educational materials for patients, relatives, and health-care workers.^(28)^ Regarding the ICU, some E-programs were tested to enhance the adequate recovery of critically ill patients,^(23)^ reducing psychological damage in their surrogates.^(18,22,29)^ Nguyen et al.,^(29)^ studying 169 surrogates, demonstrated that satisfaction with ICU care (OR = 1.39 [95%CI 0.69 - 25.77]) or medical information provision (OR = 0.82 [95%CI 0.3.75]) and the presence of anxiety (OR = 1.05 [95%CI 0.97 - 1.13]) or depression symptoms (OR = 1.03 [95%CI 0.95 - 1.12]) were not associated with internet use. Mistraletti et al.^(18)^ studied 332 relatives and showed that an information brochure and website designed to solve relatives’ needs improved family members’ comprehension (about prognosis [from 69 to 84%, p = 0.04] and about therapeutic procedures [from 17 to 28%, p =0.03]) and reduced their prevalence of stress symptoms (Poisson coefficient = −0.29 [ −0.52 to −0.07]). In our study, only 11.5% of the subjects’ relatives accessed the website; however, these family members presented higher satisfaction with care and a lower prevalence of clinical anxiety (but not depression) than surrogates who did not access it.

The main strength of this study is the heterogeneity of the studied population. This approach could guarantee the generalizability of the communication tools, which were specifically designed for this purpose. The tool also seemed easy for the staff to use without increasing their workload, only informing relatives about the existence of the website. However, this study has important limitations. First, even if the intervention was effective, only 11.5% of relatives visited the website. This low proportion may be due to the lack of familiarity of people with the internet, the low educational level of relatives, or the lack of attractiveness of this kind of educational method. Second, the sample size was relatively small and comprised a selected population, although this was a multicenter study. Third, the analysis was limited to only a few days after ICU admission and does not provide information about long-term psychiatric symptoms. Last, it is an observational study, and this design limits the ability to conclude whether the differences in outcomes were a result of the intervention. Randomized trials are needed to explore the potential of educational strategies to support family members in ICUs with flexible visiting hours.

A better understanding of the information needs of critically ill patients’ proxies may help physicians improve their medical information delivery and encourage them to discuss the proxies’ internet searches with them, avoiding reactions perceived as negative by proxies. Our data showed that access to an educational website was associated with less anxiety and greater satisfaction among family members of ICU patients. Therefore, in the era of widespread health-related internet use, physicians should take into account the fact that the majority of the families of critically ill patients seek medical information online. The development of structured tools with standardized and adequate information can be very useful in relieving the stress and anxiety of relatives of critical patients, thus becoming an ally for information exchange and improved communication between the ICU staff and their patients and relatives.

## CONCLUSION

Access to an educational website designed for family members of critically ill patients was associated with higher satisfaction with care and a lower prevalence of clinical anxiety; however, this association may not be causal.
